# Heritability of distichiasis in Havanese dogs in Norway

**DOI:** 10.1186/s40575-021-00110-5

**Published:** 2021-11-16

**Authors:** Kim K. L. Bellamy, Frode Lingaas, Per Madsen

**Affiliations:** 1 Department of Preclinical Sciences and Pathology, Faculty of Veterinary Medicine, Norwegian University of Life Sciences, P.O. Box 369, sentrum, N-0102 Oslo, Norway; 2 The Norwegian Kennel Club, P.O. Box 52, Holmlia, 1201 Oslo, Norway; 3 Center for Quantitative Genetics and Genomics, Aarhus University, dk-8830 Tjele, Denmark

**Keywords:** Distichiasis, Havanese, Heritability, Prevalence

## Abstract

**Background:**

Distichiasis is a presumed inherited eyelid disease, characterized by misplaced eyelashes. The effect on eye health and animal welfare varies between individuals; most mild cases show no clinical signs, but some affected animals develop painful corneal disease.

In this study, we investigated the prevalence and heritability of distichiasis in the Norwegian population of Havanese dogs.

**Results:**

A total of 1156 Havanese were included in the study. Out of these, 168 were affected with distichiasis, making the prevalence in our sample 14.5% (95% CI 12.5–16.6%). There was no sex predisposition. Most affected individuals were graded “mildly affected”.

The estimates generally showed high heritabilities, which varied between 0.276 (linear model) and 0.720 (Bayesian threshold model). The linear estimates, after conversion to the underlying scale (h^2^_l_ = 0.664–0.674), corresponds well to the results of the Bayesian models.

**Conclusions:**

The estimated heritability of distichiasis in Havanese is high and the prevalence is moderate. The high heritability indicate that a significant selection response could be obtained by simple mass selection. To secure good animal welfare, it’s important to control the number of affected individuals and especially the severely affected.

## Plain English summary: prevalence and heritability of distichiasis in a population of Havanese Dogs

Distichiasis is an eye condition, characterized by misplaced eyelashes, that is frequently seen in dogs. Some dog breeds appear to be more at risk than others. The degree of clinical signs in affected dogs varies a lot. Many mild cases appear to be completely asymptomatic, while others suffer pain and damage to the eye, which necessitates removal of the hairs.

In this study, we investigate both how common distichiasis is in the Havanese dog breed and estimate the degree of genetic influence on the trait. We find that 14.5% of eye screened Havanese, registered in the Norwegian Kennel Club, are affected with distichiasis. Most cases are graded “mild”. There is no significant difference in how many males and females are affected.

We find high heritability estimates of distichiasis in Havanese (≈0.28 calculated by linear models and 0.59-0.72 calculated by Bayesian threshold models), showing a high genetic influence on the trait. The high estimated heritability mean that it should be possible to reduce the prevalence of the condition, and contribute to improved animal welfare, though systematic breeding.

We recommend that all Havanese are eye screened prior to breeding, to control the prevalence of distichiasis, as well as other eye conditions that are relevant in the breed, like cataracts. Dogs with severe distichiasis or ectopic cilia should not be bred. Dogs with mild or moderate distichiasis may be bred to an unaffected partner.

## Background

Distichiasis is a condition characterized by misplaced eyelashes. The term distichiasis stems from the Greek words di and stichos, meaning two rows. In dogs the term in somewhat misleading, as there is no complete row of extra lashes, but rather one or several individual stray hairs [1, 2].

The hairs arise from ectopic hair follicles in the tarsus and emerge through the meibomian duct openings [2, 3]. The condition may be uni- or bilateral and the number of misplaced cilia vary considerably between eyes and between individuals [3, 4]. The clinical relevance is variable. Many affected individuals show no clinical signs, but some dogs experience corneal damage and pain that requires removal of the hairs [1, 2, 5]. The degree of pain and corneal damage vary and are not directly proportional to the number of cilia [4].

In some cases, one or a few single hairs grow through the palpebral conjunctiva a few millimeters from the eyelid margin, directly onto the cornea. These hairs are referred to as ectopic cilia [1–3, 5]. Distichiasis and ectopic cilia are two different forms of disease that are both caracterized by misplaced eyelashes and are grouped together in the ECVO (European College of Veterinary Ophthalmologists) certificates. Ectopic cilia generally cause significant corneal disease and pain [1, 3, 5].

Clinical signs of distichiasis and ectopic cilia may include epiphora, squinting of the eyes, photophobia, keratitis, and corneal damage. Because many dogs affected with distichiasis don’t show clinical signs, it’s important to rule out possible additional diagnoses when clinical signs are evident [3].

Distichiasis and ectopic cilia normally occur early in life and are often congenital, but may develop at any age [4, 5]. According to the European College of Veterinary Ophthalmologists (ECVO) scheme, a dog is considered to be “affected” by distichiasis if the diagnosis has been made by a panel member once, even if no stray cilia are detected on subsequent examinations [5].

Both distichiasis and ectopic cilia are classified as presumed hereditary eye diseases [5]. Studies show evidence of a genetic component [6] and that affected dogs are more likely than unaffected dogs, to parent affected offspring [7].

Some breeds, like Pekingese, poodles and both American and English cocker spaniels, are reported to be affected more frequently than others [3, 4, 8], which support that the trait is heritable. Heritability estimates for distichiasis are high in cocker spaniels [7, 9], but low in Tibetan terriers [10]. Distichiasis is reported in Havanese through the open databases of kennel clubs in several countries, which indicate that the problem is widespread in this breed.

In this study, we calculate heritability estimates for distichiasis in Havanese with ECVO eye results registered in The Norwegian Kennel Clubs (NKK) database. Increased knowledge of the genetic component of the trait would be important to select an optimal breeding strategy, to secure a low frequency of the trait and good animal welfare.

## Results

### Prevalence and grading

Of the 1156 dogs in the material, 168 were affected with distichiasis, making the prevalence of distichiasis in our sample 14.5% (95% CI 12.5–16.6%).

Of the affected dogs, 86.9% (*n* = 146) were graded “mild”, 10.1% (*n* = 17) were not graded, 2.4% (*n* = 4) were graded “moderate” and 0.6% (n = 1) was graded “severe”. Of the graded dogs, 96.7% were graded “mild”, 2.6% were “moderate” and 0.7% were graded “severe”. Ectopic cilia were noted in a single dog.

### Heritability estimates

The heritability was estimated using both linear and Bayesian threshold models.

### Linear model

Alternative linear models, with slight differences in how year of diagnosis was treated, all gave heritability estimates around ~ 0.28 (Table 1).

**Table 1:** Estimated variance components and heritability, with standard deviation in brackets

Model	Genetic variance	Residual variance	Heritability
1	0.034 (0.007)	0.088 (0.006)	0.276 (0.050)
2	0.034 (0.007)	0.087 (0.006)	0.279 (0.051)
3	0.034 (0.007)	0.088 (0.006)	0.280 (0.051)

### Bayesian threshold models

Heritability estimates using a Bayesian threshold method, were 0.594, 0.720 and 0.674 for the three models, respectively.

**Fig. 1:**
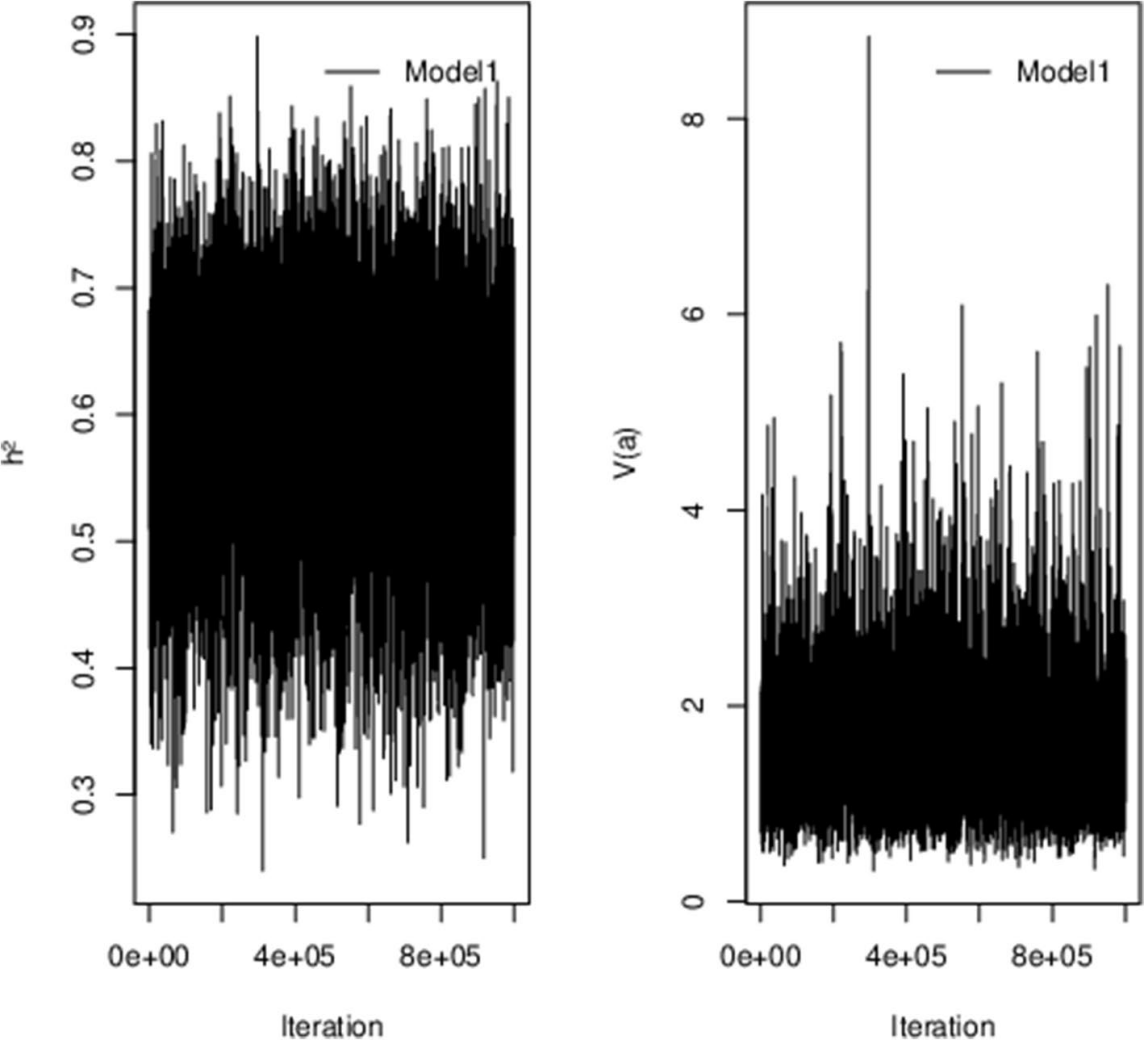
Trace plots for heritability (h^2^) and genetic variance (${\sigma}_a^2$) from model 1

Posterior means, highest posterior density (HPD) regions and effective sample size for genetic variance and heritability from the three models are shown in Table 2. Trace plots for heritability and genetic variance for model 1 are shown in Fig. 1 (trace plots for model 2 and 3 are similar and not shown).

**Table 2:** Posterior means and highest posterior density (HPD) regions for dispersion parameters

Model	Genetic variance (${\sigma}_a^2\Big)$				Heritability			
	Posterior mean	HPD region		Effective sample size	Posterior mean	HPD region		Effective sample size
		Lower	Upper			Lower	Upper	
1	1.598	0.578	2.890	1455.0	0.594	0.415	0.762	1535.0
2	3.078	0.761	6.407	496.8	0.720	0.540	0.900	459.0
3	2.373	0.686	4.652	707.5	0.674	0.491	0.854	991.9

### Comparison of the heritability estimates from the linear- and Bayesian threshold models

To compare the heritability estimates from the linear models with the estimates from the Bayesian threshold models, the results of the linear models were converted to the underlying normally distributed liability scale, as described by Dempster and Lerner [11] (Table 3).

**Table 3:** Heritabilities from the linear models on the observed (*h*^*2*^_*o*_*)* and underlying liability scale (*h*^*2*^_*l*_) with standard error in brackets and heritabilities from the threshold models (*h*^*2*^_*t*_) with highest posterior density region in brackets

Model	*h*^*2*^_*o*_	*h*^*2*^_*l*_ *p* = 0.145 = > *z*_*ord*_ = 0.227	*h*^*2*^_*t*_
1	0.276 (0.050)	0.664 (0.120)	0.594 (0.415–0.762)
2	0.279 (0.051)	0.671 (0.123)	0.720 (0.540–0.900)
3	0.280 (0.051)	0.674 (0.123)	0.674 (0.491–0.854)

The relatively small data set leads to large standard errors for the heritabilities estimated by the linear models, as well as large HPD regions for the estimates from the threshold models.

### Correlation of breeding values

The correlation between breeding values estimated by the linear models and breeding values estimated by the Bayesian threshold models was high (> 0.97) (Table 4). Spearman rank correlation > 0.98 indicates almost identical ranking based on breeding values predicted by linear and threshold models.

**Table 4:** Correlation coefficients between breeding values calculated by a linear model and breeding values calculated by a threshold model

Model	Pearson’s correlation	Spearman’s rank correlation
1	0.984	0.993
2	0.976	0.984
3	0.980	0.988

### Sex effects

The 1156 dogs consisted of 426 males and 730 females. There was no significant difference in prevalence between males (13.1%) and females (15.3%) (*X*^2^(1, *N =* 1156) = 1.05, *p* = .307).

The genetic correlation between the estimates analyzed within sex were 0.885 (0.144), 0.934 (0.139) and 0.930 (0.141) for model 1–3 respectively, which means there is no significant effect of sex on the trait.

### Conflicting results and age

By comparing the distichiasis result of the first and last examination in dogs that have been eye screened at least twice, we found that out of the 1156 Havanese in our material, 49 had gone from unaffected to affected, and 11 had gone from affected to unaffected, in the first and last examination respectively. Out of the 2259 eye certificates registered in the kennel club database between 2005 and 2020, only 33 came from dogs that were younger than 1 year old at the time of examination.

### Test percentage

By comparing registration numbers and the number of ECVO eye certificates in the kennel club database from 2005 to 2020, we find a test percentage of 21.3%.

Out of all litters registered between 2005 and 2020, at least one puppy was eye screened in 48.8% of them.

## Discussion

Our results show a prevalence of distichiasis in Havanese of 14.5%. To the authors knowledge, few studies have been conducted on the prevalence of distichiasis in different dog breeds, which gives little reference for comparison. In a study of Tibetan terriers, 11.43% of the study population were affected with distichiasis [10], while a study of English cocker spaniels in Denmark showed a prevalence of distichiasis of 49.31% [7]. Havanese have a long and furnished double coat. Considering that most of the predisposed breeds, like poodles, Pekingese and cocker spaniels are also heavily coated, it’s possible that selection for a profound coat could contribute to an increased risk of distichiasis.

Since April 2016, the Norwegian Kennel Club require a valid ECVO certificate for all Havanese used for breeding, for the offspring to be eligible for registration [12]. Due to breed club recommendations [13], which have been in place since the breed club was founded in 2009, most breeding animals were eye examined yearly prior to 2016 as well. The number of individual Havanese that have been eye screened at least once between 2005 and 2020, equals 21% of the number of Havanese registered in the same time period. The number of individual Havanese litters, in which at least one puppy has been eye screened, equals 49% of the number of litters registered in the same time period. Based on these numbers, we consider the test-percentage sufficient.

As there is no official dog registry in Norway, it is uncertain what percentage of purebred dogs are registered in the Norwegian Kennel Club. However, by comparing numbers from a microchip registry to the kennel club database, we find that ≈70% of microchipped dogs are registered in the NKK. Because the total number of microchipped dogs also include mixed breeds, it’s reasonable to assume that more than 70% of purebred dogs in Norway are registered in the kennel club.

More females than males are eye screened, and we presume this is because more females than males are used for breeding. This indicates that dogs that are intended for breeding are eye screened more often than other dogs. Breeders often keep and test one or two puppies from each litter with the intention of continuing their breeding program. We therefore believe that a large portion of the active breeding population is tested, as opposed to certain lines or litters being overrepresented.

Based on these factors, we believe our material is representative of Havanese registered in the Norwegian Kennel Club and the Norwegian population of Havanese in general.

We have considered the possibility of owners removing misplaced hairs prior to examination, which could result in misclassification of affected dogs, but based on our knowledge of the breed community we believe this to be unlikely. Because a distichiasis diagnosis does not exclude a dog from breeding, the owner’s motivation to falsify the result of the examination is limited. We also believe that potential removal of hairs in a limited number of dogs, would be relatively equal in different breeding lines/families, thus we believe the potential effect on heritability estimates is neglectable. However, the “once affected=always affected” policy, is established to limit this source of error.

In a total of 60 dogs, the distichiasis status changed from the first to the last eye examination. In 49 dogs the result went from unaffected to affected and in 11 dogs the results went from affected to unaffected, from the first to last examination respectively. The 49 dogs that went from unaffected to affected, could have developed distichiasis later than usual, or it’s possible that a few very mild cases may have gone undetected in the first examination. However, according to the ECVO “once affected=always affected” policy, no dog should go from affected to unaffected with distichiasis. The 11 cases could be caused by human error in filling out the certificates and we consider the number low enough not to represent an important source of error.

Distichiasis normally occurs early in life and is often congenital. However, if a large portion of dogs were tested at a very young age, it could potentially result in an underestimation of prevalence. In our material, only 33 out of 2259 eye certificates came from dogs that were under 1 year old at the time of examination, which indicate that this source of error is most likely neglectable.

Our results show high heritability estimates for distichiasis in Havanese dogs, using both linear and Bayesian threshold models. This means that it should be possible to control the prevalence of the disease through traditional mass selection, without complex routines for index estimation.

Transformation of the heritabilities estimated by the linear models to the underlying liability scale, show results that are similar to the Bayesian estimates. Additionally, for all three models there are very high correlations (> 0.97) between breeding values calculated by the linear models and breeding values calculated by the Bayesian threshold models. Our results indicate that with prevalence as in the present data, computationally heavy Bayesian threshold models could be successfully substituted by linear models.

Most of the affected individuals in our material were graded mild, with comments often indicating that only one or a few cilia were present. The hairs are often soft, which may partly explain why many Havanese affected with distichiasis don’t show clinical signs [1, 14]. However, as we know distichiasis and ectopic cilia cause pain and corneal damage in some individuals, measures should be made to control the prevalence.

The ECVO breeding guidelines states that it is “optional” to breed affected animals, with the exception of severe cases [5]. Mild and moderate cases may only be bred to an unaffected partner [15]. Results from Petersen et al. [7] supports this recommendation, as the risk of producing affected offspring is higher when two affected dogs are bred, than when an affected animal is paired with an unaffected partner. It is further recommended to exclude all dogs affected with ectopic cilia [15]. Because the number of severely affected Havanese is very low, this policy will exclude very few dogs from breeding and at the same time prevent high risk combinations.

Our findings support that the mandatory ECVO eye screening prior to breeding should be continued. Results from routine eye screenings can be helpful in monitoring the prevalence of distichiasis, since they are easily available through the NKK open database. The ECVO eye screening scheme is highly standardized and good routines are implemented for secure identification of animals and publication of results. This makes it a valuable breeding tool, to help breeders reduce the prevalence of distichiasis as well as other eye diseases that are relevant in the breed, like cataracts [16, 17].

## Conclusion

We show that 14.5% of Havanese that are registered in the Norwegian Kennel Club and have been eye screened between 2005 and 2020, are affected with distichiasis.

The heritability estimates for the disease are generally high: around 0.28 calculated by linear models, which is comparable to the values from the Bayesian threshold models of 0.59–0.72, after conversion to the underlying liability scale. The high heritability suggest that it should be possible to reduce the prevalence of distichiasis through routine eye screenings and traditional mass selection.

Dogs that have ectopic cilia or severe distichiasis should be excluded from breeding, while dogs with mild or moderate distichiasis may be bred to an unaffected partner if they have other valuable traits that may be beneficial for the breed.

## Methods

### Dogs

The study material was collected from The Norwegian Kennel Clubs (NKK) database. For an eye certificate to be registered in the NKK database, thorough protocols must be followed to secure quality assurance of the diagnostic testing. Prior to examination, owners must consent to the result being made publicly available. Animals are identified by microchip numbers that are linked to the kennel club registry and controlled by the examiner prior to examination. Only veterinarians who are certified eye scheme examiners and have completed the Nordic Eye Examination Committees extensive educational program [18], can register results in the database.

The Havanese breed was selected for this study because it is the most registered breed in the Companion and Toy group in The Norwegian Kennel Club, and one out of two breeds in the top 16 most registered breeds were a yearly eye examination is mandatory prior to breeding. Between 2005 and 2020, a total of 5422 Havanese, from 1756 different litters, were registered in the Norwegian Kennel Club [14].

The high registration numbers, high frequency and quality of diagnostic testing, as well as availability through an open database, entailed good quality data was available for analysis.

Inclusion criteria were Havanese that are registered with a pedigree in the NKK and have at least one ECVO certificate registered between 2005 and 2020. The material was readily available from the NKK open database and all available certificates were included (prior to duplicate removal and removal of dogs with missing information).

The classification of dogs as either “affected” or “unaffected” with distichiasis is done by visual inspection by a certified eye scheme examiner, as described in the ECVO manual [5], and the presence or absence of distichiasis is mandatory to record in all examinations. The diagnosis is classified as either “affected” or “unaffected”, but the examiner may also grade the diagnosis. In patients were signs of corneal irritation, ectopic cilia and/or hard and stiff distichia are present, the grade is always classified as “severe” [5]. Because there was little variance in the grading in our material, and grading is not mandatory, we classified the dogs as “affected” or “unaffected” in the analysis.

Because dogs are often examined more than once during their lifetime, duplicate observations were removed. From an original sample of 2259 observations, 1166 unique Havanese remained after duplicate removal. The ECVO scheme states that once a dog is determined to be “affected” with distichiasis by a panel member, the diagnosis is final, i.e. once affected = affected [5]. We classified our sample accordingly, by keeping the “worst” diagnosis in individuals with conflicting results.

Out of the 1166 dogs that remained after duplicate removal, 10 were removed because the date of examination (*n* = 7) or date of birth (*n* = 3) was missing. The remaining 1156 dogs came from 857 different litters.

Tracing of the 1156 Havanese in the Norwegian Kennel Clubs pedigree files, resulted in a pedigree file of 3327 dogs.

### Heritability estimates

#### Linear models

Three different linear models were used:

1 $$y= Sex+ Age+a+e$$

2 $$y= Sex+R\_ Year+ Age+a+e$$

3 $$y= Sex+R\_ Year+b\ast C\_ Age+a+e$$

where: *y* = vector of observed diagnoses, *Sex* = fixed effect of sex, *Age* = fixed effect of age in years, *R_Year* = year of diagnosis, *b* = regression on *C_Age*, where *C_Age* is age at diagnosis as a continuous variable, *a* = random additive genetic effect and *e* = the random residual.

Assumption for random effects are:

- $a\sim N\left(0,{\sigma}_a^2A\right)$, where ${\sigma}_a^2$ is the genetic variance and *A* is the additive relationship matrix
- $e\sim N\left(0,{\sigma}_e^2I\right)$, where ${\sigma}_e^2$ is the residual variance and *I* is an identity matrix.

The analysis was conducted with the average information restricted maximum likelihood (AI-REML) module in DMU [19].

#### Bayesian threshold models

The same three models were analyzed by a Bayesian threshold model, using the Gibbs Sampler.

module in DMU [19]. To ensure identifiability of dispersion parameters and threshold, the residual variance (${\sigma}_e^2$) was restricted to unity. Because only one registration was included per individual, which is known to create problems in threshold animal models, the genetic variance was sampled based on individuals that have offspring, as described by Ødegård et al. 2010 [20].

For each of the 3 models, the Gibbs Sampler was run for 1,100,000 rounds with the first 100,000 discarded as burnin. Every 10’th of the remaining 1,000,000 samples was stored for the Post Gibbs analysis.

Post Gibbs analysis was conducted by BOA software [21] and own developed software for computation of effective sample size.

For each of the stored samples, heritability was computed as $\frac{\sigma_a^2}{\sigma_a^2+1}$.

Mixing properties of the Markov chain Monte Carlo (MCMC) chains were visually inspected by trace plots.

### Comparison of the heritability estimates from the linear and Bayesian threshold models

The heritabilities from the threshold models are expressed on the underlying scale. For comparison with the results from the linear model, the estimated heritabilities from the linear models were converted to the underlying normally distributed scale by the formula by Dempster and Lerner [11]:

$${h^2}_l=\left({h^2}_ox\ p\ x\ \left(1-p\right)\right)/{z^2}_{ord}$$

where:

*h* ^*2*^_*l*_ = heritability on the underlying scale.

*h* ^*2*^_*o*_ = heritability on the observed scale.

*p* = frequency.

*z*_*ord*_ = height of the standard normal distribution at the threshold value corresponding to *p.*

### Correlation of breeding values

The correlation between estimated breeding values from the linear and Bayesian threshold models were calculated as both Spearman and Pearson correlation coefficients.

### Sex effects

To correct for potential sex effects and possible confounding with age, both age and sex were included in the models used for the heritability estimates (model 1–3).

Sex effect on diagnosis was investigated by running a bivariate restricted maximum likelihood (REML) analysis on the three models from the heritability estimates (containing age-effect), treating diagnosis in each sex as two separate traits.

The significance level for potential difference in prevalence between males and females, were calculated using the chi-squared test.

## Data Availability

The phenotype- and pedigree information that support the findings of this study is freely available through The Norwegian Kennel Clubs open database, Dogweb [14].

## Abbreviations

AI-REML: Average information restricted maximum likelihood
CI: Confidence interval
ECVO: European College of Veterinary Ophthalmologists
HPD: Highest posterior density
MCMC: Markov chain Monte Carlo
NKK: The Norwegian Kennel Club
REML: Restricted maximum likelihood
